# CompCytogen of two of the smallest Amazonian fishes: *Fluviphylax simplex* Costa, 1996 and *Fluviphylax zonatus* Costa, 1996 (Cyprinodontiformes, Poeciliidae)

**DOI:** 10.3897/CompCytogen.v5i5.1562

**Published:** 2011-12-22

**Authors:** E.R. Souza, L.B. Ribeiro, E. Feldberg, I.P. Farias T. Hrbek, M.C. Gross

**Affiliations:** 1 Laboratório de Evolução e Genética Animal, Instituto de Ciências Biológicas, Universidade Federal do Amazonas, Manaus, Amazonas, Brazil; 2 Laboratório de Genética Animal, Instituto Nacional de Pesquisas da Amazônia, Manaus, Amazonas, Brazil; 3 Laboratório de Citogenômica Animal, Instituto de Ciências Biológicas, Universidade Federal do Amazonas, Manaus, Amazonas, Brazil

**Keywords:** Chromosomes, heterochromatin, killifish, meiosis, mitosis

## Abstract

The genus *Fluviphylax* Whitley, 1965is comprized of five valid species (*Fluviphylax pygmaeus* Myers et Carvalho, 1955, *Fluviphylax zonatus*, *Fluviphylax simplex*, *Fluviphylax obscurus* Costa, 1996,and *Fluviphylax palikur* Costa et Le Bail, 1999), which are endemic to the Amazon region. These fishes are the smallest known South American vertebrates and among the smallest know vertebrates on Earth. All species but the type *Fluviphylax pygmaeus* have been described in late 1990’s, and much remains unknown about the biology, taxonomy and systematics of this group of fishes. The aims of the present study were to establish the diploid and haploid number of *Fluviphylax zonatus* and *Fluviphylax simplex*, and to find species-specific markers for the discrimination of taxa. The diploid number for both species was 48 chromosomes, with no sex chromosome heteromorphism. *Fluviphylax zonatus* exhibited the karyotypic formula 4m+8sm+22st+14a and FN=82, and *Fluviphylax simplex* exhibited 4m+16sm+18st+10a and FN=86. The determination of the total mean length of the chromosomes and their grouping into five size classes demonstrated different chromosome composition of the two species. This difference was further supported by the distribution of constitutive heterochromatin. The meiotic analysis revealed 24 bivalents in both species, but *Fluviphylax zonatus* exhibited chromosomes with late pairing of the telomeric portions in the pachytene. These data reveal that cytogenetic characterization is useful and important for the discrimination of these species. Our study further indicates that this method could be employed in the analysis of other species of small fishes that are difficult to distinguish using traditional morphological traits or are morphologically cryptic.

## Introduction

The Amazonian region has the most diverse freshwater fish fauna in the world, which, although only imperfectly known (Santos and Ferreira 1999). In general, cytogenetic studies of freshwater Neotropical fishes have resulted in the analysis of approximately 1040 species, of which more than 70% correspond to the orders Characiformes and Siluriformes (Oliveira et al. 1988, 2007, 2009). Cyprinodontiform fishes comprise approximately 850 species, mostly Neotropical fishes, of which only 67 neotropical species have cytogenetic information (Costa 1998, Oliveira et al. 2007, 2009). This dearth of information from cytogenetic data is mainly due to the low commercial importance and the small size of specimens that make up the cyprinodontiforms, limiting our understanding of their chromosomal organization and karyotype evolution.

The cyprinodontiforms are a large and diverse group of teleostean fishes comprising the family Poeciliidae. The fishes of the family Poeciliidae are small and laterally compressed, widely distributed in American and African continent. The Poeciliidae include the subfamilies Poeciliinae, Aplocheilichthyinae and Procatopodinae, a group composed of the South-American *Fluviphylax* Whitley, 1965 and the African procatopodines (Ghedotti 2000, Reis et al. 2003). The cyprinodontiform genus *Fluviphylax* comprises five species: *Fluviphylax pygmaeus* Myers et Carvalho, 1955, *Fluviphylax zonatus* Costa, 1996, *Fluviphylax simplex* Costa, 1996, *Fluviphylax obscurus* Costa, 1996 and *Fluviphylax palikur* Costa et Le Bail, 1999 (Myers 1955, Costa 1996, Costa and Le Bail 1999). These fishes are commonly known as killifish and are the smallest South American vertebrates, reaching a maximal size of 22 mm. The genus is endemic to the basins of the Amazon and Orinoco Rivers and Atlantic drainages of the state of Amapá, Brazil (Myers 1955, Costa 1996, Costa and Le Bail 1999, Arrington and Winemiller 2003, Hoeinghauset al. 2004, Lasso et al. 2004). Species *Fluviphylax simplex* and *Fluviphylax zonatus* are endemic to the central portion of the Amazon River Basin. The geographic distribution of *Fluviphylax simplex* is in the Amazonian floodplain (Várzea) from the Amanã Reserve to the city of Santarém, whereas *Fluviphylax zonatus* is restricted to the lower Negro River (Costa 1996, Souza 2008).

The taxonomic history of the genus *Fluviphylax* is relatively recent. The first species was discovered and scientifically described by Myers and Carvalho in 1955. The genus *Fluviphylax* has paucity of systematic, taxonomic and genetic information, with our knowledge being almost entirely restricted to the information published in the original description. Therefore, cytogenetic studies significantly expand our knowledge base of this group, especially in the realm of understanding of chromosome evolution of *Fluviphylax*.

Cytogenetic studies have contributed significantly to the identification of fishes (Nakayama et al. 2001, 2008, Teixeira et al. 2006) as well as the understanding of chromosome evolution in Amazonian ichthyofauna (Benzaquem et al. 2008, Gross et al. 2009). However, such studies have been restricted to larger species that are more easily handled. The present study reports a cytological characterization of two species of *Fluviphylax*, obtained by modifications of the technique described by Moreira-Filho and Bertollo (1990) and karyotype comparison with other species Poeciliidae.

## Materials and methods

**Figure 1.**
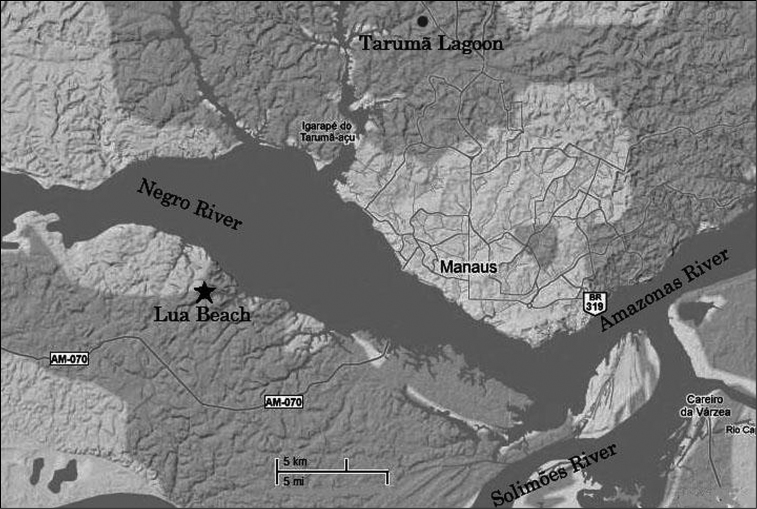
Sampling locations circle and star indicate sampling points for *Fluviphylax zonatus* and *Fluviphylax simplex*, respectively

**Figure 2.**
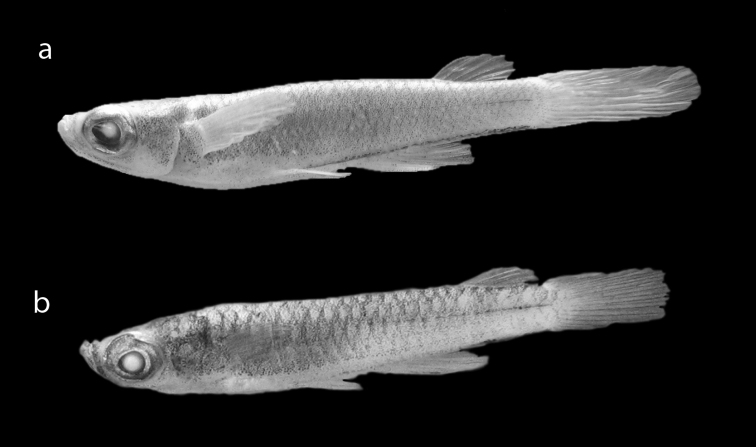
**a** *Fluviphylax zonatus* with 20.0 mm SL **b** *Fluviphylax simplex* with 18.4 mm SL.

Specimens of *Fluviphylax simplex* were collected from Lua Beach (3°07'31.7"S, 60°10'38.9"W) near the confluence of the Negro and Solimões Rivers, Amazonas, Brazil. Specimens of *Fluviphylax zonatus* were collected from a small lake (3°00'19.2"S, 60°03'22.6"W) located near Manaus that gathers water from the Tarumã River, which is a tributary of the Negro River (Figs 1, 2). We analyzed 24 specimens of the *Fluviphylax simplex* and 37 of the *Fluviphylax zonatus*. The gender determination was made only for adults specimens of each species being 6 males, 8 females and 10 indeterminated for *Fluviphylax simplex*, and 7 males, 6 females and 24 interminated for *Fluviphylax zonatus*. Collections were performed under a license from the Brazilian Institute of the Environment and Renewable Natural Resources (IBAMA n. 11325-1/2007). Following the chromosome preparation, some specimens were fixed in 95% alcohol. Voucher specimens were deposited in the Fish Collection at the Instituto Nacional de Pesquisas da Amazônia (INPA) in Manaus, State of Amazonas, Brazil (number 25527), and in the Animal Genetics Tissue Collection of the Laboratory of Animal Evolution and Genetics of the Institute of Biological Sciences of the Universidade Federal do Amazonas (Brazil).

Due to the small size of the specimens (less than 20 mm in total length), the cell preparations were obtained through the maceration of the each individual in a cuvette containing 6 ml of hypotonic KCl solution with the aid of two pairs of tweezers. Eyes and intestines were removed prior to maceration. The cell suspension was infused with 0.3 ml of 0.0125% colchicine solution. This preparation was incubated for 40 minutes at 37°C. The subsequent fixation of cells was carried out following the method of Moreira-Filho and Bertollo (1990). C-banding was used to characterize the constitutive heterochromatin distribution (Sumner 1972).

The chromosome preparations were analyzed under an optical microscope with an immersion objective. Selected cells were photographed with a Canon Power Shot A650 IS digital camera. The mounting of karyotypes was carried out with mitotic metaphase chromosomes, which were cut out and tentatively paired. The chromosomes were measured using the free ImageJ program and organized in decreasing order of size. Chromosome morphology was determined taking into account the position of the centromere, based on the method proposed by Levan et al. (1964). Chromosomes were classified as metacentric (m), submetacentric (sm), subtelocentric (st) and acrocentric (a) (Levan et al. 1964). The fundamental number (FN) was determined based on the number of chromosome arms, considering metacentric, submetacentric and subtelocentric chromosomes as having two arms and acrocentric chromosomes as having only one arm. Using the data of the mitotic chromosome measurements, of all karyotype for both species, a comparative analysis between *Fluviphylax simplex* and *Fluviphylax zonatus* was performed based on the length frequency of chromosome pairs by size class. Sturges’ formula was used for determining the ideal number of classes: n = 1+3.32*LogN, in which “n” is the number of classes and “N” is the number of chromosomes in the haploid complement (Fonseca and Martins 1982).

## Results

In this study we analyzed 428 cells of the *Fluviphylax zonatus*, 16% corresponded to mitotic metaphase cells and the others were to meiotic cells, of which 46% leptotene/zygotene, 24% pachytene, 24 % diplotene/diakinesis/metaphase I and 6% in metaphase II. For *Fluviphylax simplex* wereobtained 384 cells corresponded to 36% mitotic cells in metaphase and the others were to meiosis cells, of which 12% leptotene/zygotene, 67% pachytene, 16 % diplotene/diakinesis/metaphase I and 5% in metaphasee II.

**Figure 3.**
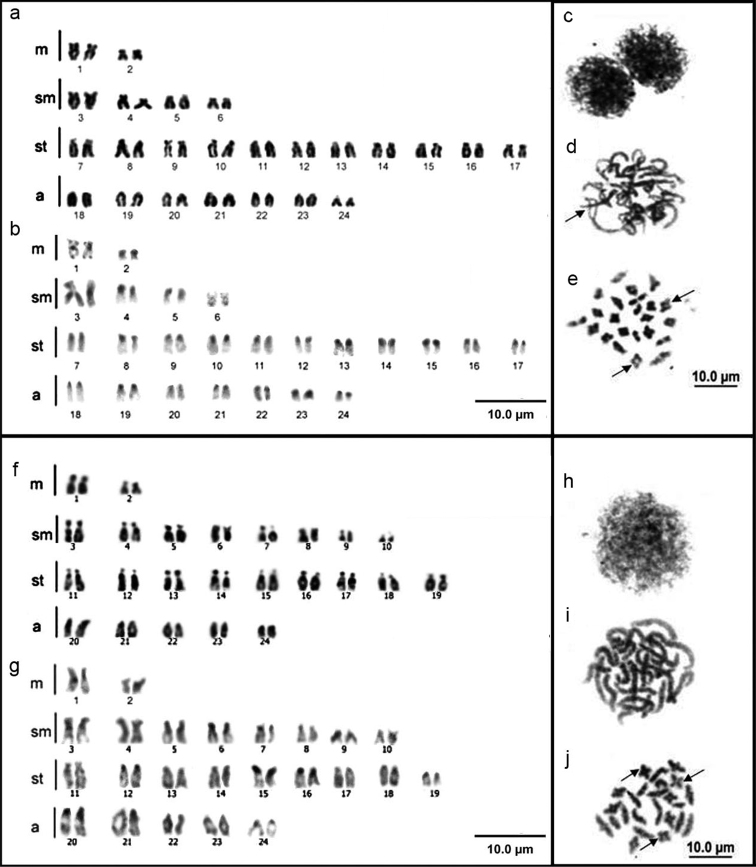
Data on *Fluviphylax zonatus* and *Fluviphylax simplex*: with conventional staining **a, f** C-banding **b, g** initial leptotene stage **c, h** pachytene stage revealing 2n=24II **d, i** arrow indicates late pairing in some telomeric regions **d** diplotene stage with 2n=24II, arrows indicate bivalents with interstitial chiasma; arrow indicates bivalents with terminal chiasma **e, j**

**Figure 4.**
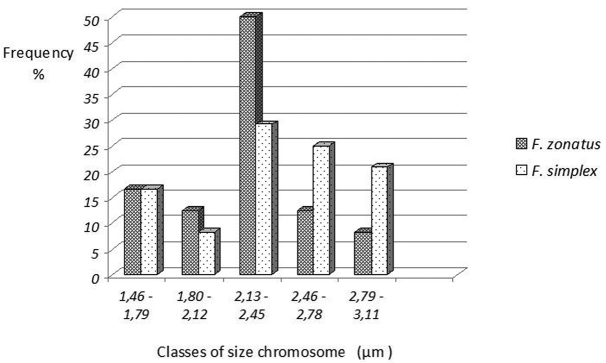
Analysis of chromosome size in *Fluviphylax zonatus* and *Fluviphylax simplex*; Y axis gives frequency of chromosomes with pair sizes in classes informed on X axis.

In the mitotic analysis, both species had a diploid number of 48 chromosomes, with symmetrical karyotypes and no sex chromosome heteromorphism. *Fluviphylax zonatus* karyotype consists of 2n=4m+8sm+22st+14a (Fig. 3a), and *Fluviphylax simplex* 2n=4m+16sm+18st+10a (Fig. 3f). Constitutive heterochromatin was detected in the pericentromeric region in the majority of chromosomes in the two species (Figs 3b, g). However, in *Fluviphylax zonatus* the constitutive heterochromatin occupied entire short arms of all chromosomes, with the exception of 1^st^ and6^th^pairs. Also, in the 1^st^ pair the constitutive heterochromatin was bitelomeric and in the 6^th^ additional marks was found in long arms (Fig. 3b). In *Fluviphylax simplex* the heterochromatin blocks were less evident and in the 20^th^ pair were found additional interstitial marks on the long arms (Fig. 3g). The mean total length of the chromosomes ranged from 1.47 to 3.06 μm in *Fluviphylax zonatus* and from 1.46 and 3.28 μm in *Fluviphylax simplex* (Table 1). The grouping of chromosomes into five size classes, also revealed the different length chromosome composition between the two species (Fig. 4). In *Fluviphylax zonatus*, there was a greater frequency of chromosomes in Class III, which encompasses pairs ranging in size from 2.21 to 2.57 µm, and heterogeneity in chromosomal frequencies among other classes. Moreover *Fluviphylax simplex* also had greater frequency of chromosomes in Class III, however, the distribution of chromosomal frequencies among other classes were homogeneous.

Gonadal cells of *Fluviphylax zonatus* and *Fluviphylax simplex* at interphase and prophase I had no heteropicnotic regions that indicated the presence of sex chromatin (Fig. 3c, h). The chromosomal behavior in some meiotic phases of both species was similar, but differences were detected. In pachytenic cells, both species had 2n=24 bivalents, but *Fluviphylax zonatus* showed chromosomes with late pairing in the telomeric portions (Fig. 3d), which did not occur in *Fluviphylax simplex* (Fig. 3i). The analysis of diplotene cells also revealed 2n=24 bivalents in both species, but *Fluviphylax zonatus* had 10 bivalents with a terminal chiasma and 14 with an interstitial chiasma (Fig. 3e), and *Fluviphylax simplex* had 12 bivalents with a terminal chiasma and 12 with an interstitial chiasma (Fig. 3j). For both species, metaphases I had 2n=24 bivalents and metaphases II had n=24 chromosomes (data not shown).

**Table 1.** Average chromosome measurements (µm) and classifications in *Fluviphylax zonatus* and *Fluviphylax simplex* (Ch. Pair: Chromosome Pair; LA: Long arm; SA: Short arm; TL: Total length; AR: Arm ratio; CT: Chromosome type; m: metacentric; sm: submetacentric; st: subtelocentric; a: acrocentric). The LA, SA, TL are average values obtained from the measure of all karyotypes analyzed.<br/>

*Fluviphylax zonatus*							*Fluviphylax simplex*					
Ch. Pair	LA	SA	TL	AR	CT		Ch. Pair	LA	SA	TL	AR	CT
1	1.41	1.08	2.55	1.30	M		1	1.64	1.08	2.91	1.52	M
2	0.89	0.56	1.68	1.58	M		2	1.01	0.63	1.63	1.61	M
3	2.04	0.74	2.79	2.76	SM		3	2.14	0.82	3.13	2.61	SM
4	1.55	0.53	2.40	2.92	SM		4	2.12	0.71	2.84	2.97	SM
5	1.49	0.57	2.15	2.63	SM		5	1.79	0.79	2.54	2.26	SM
6	1.22	0.51	1.75	2.40	SM		6	1.68	0.68	2,33	2.46	SM
7	2.33	0.64	3.06	3.65	ST		7	1.57	0.66	2.31	2.39	SM
8	2.32	0.55	1.66	4.23	ST		8	1.30	0.53	1.93	2.43	SM
9	1.95	0.54	2.57	3.58	ST		9	1.12	0.44	1.65	2.54	SM
10	1.75	0.46	2.39	3.79	ST		10	0.72	0.31	1.46	2.33	SM
11	1.62	0.40	2.22	4.04	ST		11	2.57	0.49	3.28	5.20	ST
12	2.34	0.52	2.98	2.53	ST		12	2.66	0.49	3.21	5.38	ST
13	2.32	0.38	2.72	6.05	ST		13	2.26	0.57	2.98	3.93	ST
14	2.05	0.33	2.55	6.24	ST		14	2.29	0.60	2.92	3.80	ST
15	2.15	0.62	2.77	3.47	ST		15	2.44	0.45	3.08	5.40	ST
16	1.82	0.58	2.51	3.12	ST		16	1.99	0.61	2.72	3.27	ST
17	1.80	0.47	2.37	3.85	ST		17	2.13	0.47	2.68	4.57	ST
18	2.22	0.14	2.32	16.20	A		18	1.93	0.51	2.53	3.81	ST
19	2.28	0.15	2.50	15.16	A		19	1.78	0.55	2.34	3.26	ST
20	1.97	0.13	2.26	15.32	A		20	2.24	0.09	2.62	25.77	A
21	2.04	0.21	2.25	9.82	A		21	2.00	0.18	2.35	11.21	A
22	1.78	0.12	2.07	14.89	A		22	1.74	0.17	2.09	10.51	A
23	1.79	0.09	2.11	20.34	A		23	2.06	0.27	2.37	7.58	A
24	1.25	0.07	1.47	17.04	A		24	1.41	0.14	1.71	10.32	A

## Discussion

The Procatopodinae and their sister sub-family Poeciliinae belong to the family Poeciliidae within order Cyprinodontiformes (Ghedotti 2000). Most of the Neotropical Poeciliidae species are diploid with 48 chromosomes (Ohno and Atkin 1966, Ojima et al. 1976, Ráb 1984, Oliveira et al. 2007). This diploid number has been found in around 51% of the species currently described and it is considered modal number for the order Cyprinodontiformes (Scheel 1972, Oliveira et al. 1988, García et al. 2001). However, variations at the ploidia level have been reported, especially in the genus *Poecilia* (Oliveira et al. 1988, Sola et al. 1990, Galetti Jr and Rasch 1993, Arkhipchuk 1999). Phylogenetic and biogeographic studies of the poeciliid fishes (Hrbek et al. 2007) report *Fluviphylax* as basal group for Poeciliidae family, corroborating the modal diploid number found in this study.

Comparative analysis of chromosome size between *Fluviphylax zonatus* and *Fluviphylax simplex* revealed differences in the organization of the genome, that is reflected in difference of karyotype formulae, due occurrence of pericentric inversion rearrangements, which alter the karyotype formula without altering the diploid number.

Chromosomal rearrangements are considered an important mechanism of karyotypic differentiation in Aplocheiloidei and Cyprinodontiformes in general (Scheel 1972) and the fixation of chromosomal rearrangements can occur by different processes, such as genetic drift or meiotic drive (Völker et al. 2006). Currently, it is widely accepted that diversity in the size and organization of genomes is influenced by non-coding repetitive DNA, such as pseudogenes, retrotransposons, transposons and satellite DNA, the most part found in the heterochromatin. The characteristics of an actual genome of an organism is determined by differential epigenetic activity of mechanisms that cause either an increase or decrease in the amount of DNA in response to the surrounding environment (Leitch 2007). *Fluviphylax zonatus* and *Fluviphylax simplex* inhabit waters with different physiochemical characteristics, which in theory can influe via epigenetic mechanisms the organization of the karyotype. *Fluviphylax zonatus* is found in black waters from the Guiana Shield while *Fluviphylax simplex* occurs in white-water rivers (Costa 1996, Souza 2008). Geological and ecological differences between the habitats of the species analyzed may have driven their speciation, as they are subjected to different types of selective pressure, which may have allowed the fixation of rearrangements that resulted in the different karyotype formulas and specie-specific pattern of heterochromatin distribution.

Although not commonly performed, meiotic analyses are an extreme powerful tool for chromosomal characterization (Gross et al. 2009). Analysis of meiotic chromosomes was also of fundamental importance in the study of the species of *Fluviphylax*, as it resulted in more a thorough characterization of their chromosomes. The success of the meiotic analysis was likely due to the fact that the species analyzed reproduce throughout a large portion of their life cycle and thus are continuously producing gametes. Continuous reproduction was also reported by Roberts (1970) when analyzing populations of *Fluviphylax pygmaeus*. Moreover, late pairing was observed in the telomeric portions of some chromosomes in the pachytene stage in *Fluviphylax zonatus*. Late pairing is a species-specific marker in meiotic analyses and may either occur randomly or as a result of epigenetic mechanisms (Grewal and Jia 2007). As the species did not exhibit heterochromatin in the telomeric portions, this type of chromosome behavior is likely the result of gene regulation.

Some species of Poeciliidae have visible sex chromosome, such as the ZW/ZZ sex determining system in *Gambusia puncticulata* (Ráb 1984), *Poecilia latipinna* (Sola et al. 1990), *Poecilia formosa* (Sola et al. 1993) and *Poecilia sphenops* (Haaf and Schmid 1984) and the XX/XY system in *Poecilia reticulata* (Feichtinger 1988), or both systems in *Xiphophorus maculatus* (Gordon 1950). However, the two species of *Fluviphylax* analyzed here did not exhibit differentiated sex chromosomes.

Organisms with differentiated sex chromosomes generally display positive heteropycnotic corpuscles in the early stages of prophase I and differentiated meiotic behavior in these chromosomes (John 1990). In *Fluviphylax zonatus* and *Fluviphylax simplex*, the lack of atypical chromosome behavior in both confirms the absence of sex chromosomes. This lack of differentiated sex chromosomes is found in approximately 95% of Neotropical teleosts. The most striking characteristic with respect to the occurrence of sex chromosomes in fishes is their apparent random distribution across the phylogenetic tree of fishes, as different systems are found in closely related species of the same genus or even in different populations of the same species (Almeida-Toledo and Foresti 2001).

As evident from our and other studies (Benzaquem et al. 2008, Gross et al. 2009, Oliveira et al. 2009), chromosomal characterization is an important tool for taxonomic and karyotype evolution studies in fishes. Moreover,the comparative description of karyotype characteristics in *Fluviphylax zonatus* and *Fluviphylax simplex*,which are among the smallest known vertebrates, may be considered an innovative, pioneering approach for fishes of the Amazon region. The methodology employed in the present study could be used in the analysis of other species of small Amazonian fishes which abound in the Negro River basin, many of which are miniaturized and with unclear taxonomic boundaries, as well as assist in the understanding of karyotype evolution.
